# GDF11 ameliorated myocardial ischemia reperfusion injury by antioxidant stress and up-regulating autophagy in STZ-induced type 1 diabetic rats^1^

**DOI:** 10.1590/s0102-865020190110000006

**Published:** 2020-01-13

**Authors:** Zhou Bin, Yu Yanli, Qiu Zhen, Meng Qingtao, Xia Zhongyuan

**Affiliations:** I PhD, Department of Anesthesiology, Renmin Hospital of Wuhan University, China. Conception and design of the study, acquisition and interpretation of data, statistics analysis, manuscript writing; II PhD, Department of Anesthesiology, Renmin Hospital of Wuhan University, China. Acquisition of data, critical revision; III Master, Department of Anesthesiology, Renmin Hospital of Wuhan University, China. Analysis and interpretation of data, technical procedures, statistics analysis; IV Full Professor, Department of Anesthesiology, Renmin Hospital of Wuhan University, China. Design and supervised all phases of the study, final approval

**Keywords:** Receptors, Growth Factor, Oxidative Stress, Autophagy, Reperfusion Injury, Myocardium, Diabetes Mellitus, Rats

## Abstract

**Purpose:**

To investigate whether GDF11 ameliorates myocardial ischemia reperfusion (MIR) injury in diabetic rats and explore the underlying mechanisms.

**Methods:**

Diabetic and non-diabetic rats subjected to MIR (30 min of coronary artery occlusion followed by 120 min of reperfusion) with/without GDF11 pretreatment. Cardiac function, myocardial infarct size, creatine kinase-MB (CK-MB), lactate dehydrogenase (LDH), superoxide dismutase (SOD) 15-F2tisoprostane, autophagosome, LC3II/I ratio and Belcin-1 level were determined to reflect myocardial injury, oxidative stress and autophagy, respectively. In in vitro study, H9c2 cells cultured in high glucose (HG, 30mM) suffered hypoxia reoxygenation (HR) with/without GDF11, hydrogen peroxide (H_2_O_2_) and autophagy inhibitor 3-methyladenine (3-MA) treatment, cell injury; oxidative stress and autophagy were assessed.

**Results:**

Pretreatment with GDF11 significantly improved cardiac morphology and function in diabetes, concomitant with decreased arrhythmia severity, infarct size, CK-MB, LDH and 15-F2tisoprostane release, increased SOD activity and autophagy level. In addition, GDF11 notably reduced HR injury in H9c2 cells with HG exposure, accompanied by oxidative stress reduction and autophagy up-regulation. However, those effects were completely reversed by H_2_O_2_ and 3-MA.

**Conclusion:**

GDF11 can provide protection against MIR injury in diabetic rats, and is implicated in antioxidant stress and autophagy up-regulation.

## Introduction

The incidence of diabetes has increased rapidly in recent decades; the latest data from the International Diabetes Federation showed that the number of adult diabetic patients worldwide is more than 415 million. It has become a global public health problem, endangering human health and increasing social burden. Recent evidence-based on medical studies demonstrated that ischemic heart disease remains a major cardiovascular complication and the leading cause of death in diabetic patients^1^ ^,^ ^2^. Patients with diabetes are more vulnerable to myocardial ischemia reperfusion (MIR) injury, and the risk of post-myocardial infarction death is 2~4 times higher than that of non-diabetic individuals^3^. The pathophysiologic mechanisms of MIR in diabetes are complicated; this fact has increased awareness and has been deeply explored.

Our previously studies have shown that the exacerbated oxidative stress regulates cell signaling involved in types of cellular death in the process of diabetic MIR injury, which may be responsible for the increased vulnerability of the diabetic myocardium^4^ ^,^ ^5^. In addition, autophagy inhibition has also been proved to be an important cause^6^ ^,^ ^7^. Therefore, it is particularly urgent to discuss how to decrease oxidative stress and up-regulate autophagy in the diabetic myocardium, as this is beneficial to ameliorate MIR in diabetes and could provide prevention and therapy strategies for clinical practice.

Growth differentiation factor 11 (GDF11) is a member of the activin- transforming growth factor beta 1 (TGF-β1) superfamily, and is widely expressed throughout tissues and organs. Previous studies have demonstrated that GDF11 levels decline during aging, and that it is an effective approach to reverse age-related cardiac hypertrophy in old mice^8^. In addition, GDF11 was proven to have the function of reversing age-related skeletal muscular dysfunction^9^, attenuating impairments in cognitive function and synaptic plasticity^10^, remodeling aged mouse cerebral vasculature and enhancing olfactory neurogenesis^11^. Du *et al*.^12^ reported that long term targeted myocardial delivery of the GDF11 gene or daily intraperitoneal injection recombination of GDF11 protein in aged mice reduces heart failure, improves chronic cardiac function and enhances the proliferation of cardiac progenitor cells after myocardial IR. A recent study exhibited that exogenous GDF11 reduced acute myocardium IR injury by reducing oxidative stress and inflammation. It may have a morphologic and functional role in the recovery in the early stage of IR injury^13^; it is important to note that non-canonical TGF-β signaling was attenuated in this process. However, the effects of exogenous GDF11 on diabetic MIR injury, and their underlying mechanisms, remain to be elucidated.

In the present study, we established STZ-induced type 1 diabetes models and MIR models in *in vivo* and *in vitro studies*, respectively. We hypothesized that treatment with exogenous recombination of GDF11 could efficiently reduce MIR injury in diabetes and enable us to systematically explore its cardioprotective mechanisms.

## Methods

The experimental protocols were in accordance with the principles of Animal Care of Wuhan University (Wuhan, China), and approved by the Committee for the Use of Live Animals in Teaching and Research.

Male Sprague-Dawley rats of SPF level (weighing 250±10g, provided by SLAC JD Laboratory Animal Co., Ltd. Hunan China) were housed at 24°C, with a fixed light/ dark cycle (12 h light/12 h dark) and with ad libitum access to food and water.

### Diabetes and myocardial IR injury model

Diabetic rats were induced by a single intraperitoneal (i.p.) injection of streptozotocin (STZ, 60 mg/kg; Sigma-Aldrich; Merck KGaA, Darmstadt, Germany) as previously described and were considered diabetic with a fasting blood glucose ≥16.7 mmol/l^6^. A well-established myocardial IR injury model was used in the present study^6^ ^,^ ^7^. The IR injury model was finished by occluding the left anterior descending artery (LAD) for 30 min followed by 120 min of reperfusion. Sham-operated rats were subjected to the same surgical procedures without LAD ligation. Ischemia was confirmed by elevation of the ST segment with limb lead II, as well as discoloration of the ischemic area.

### In vivo experimental protocols

After 8 weeks of diabetes, diabetic (D) and age-matched non-diabetic (N) rats were randomly divided into 5 groups as follows: 1, N+sham (S); 2, N+IR; 3, D+S; 4, D+IR; 5, GDF11+D+IR. Exogenous recombination GDF11 (PEPROTEC, NJ, USA) was treated by i.p.at a dosage of 0.1mg/kg for a total of 4 weeks, starting 4 weeks before myocardium IR induction.

### Arrhythmia assessment

Heart rate, BP and ECG were simultaneously recorded with analysis software (AcqKnowledge, Biopac System, Goleta, USA). Ventricular ectopic activity was evaluated according to the diagnostic standards^6^ ^,^ ^7^. The ECGs were analyzed to determine the incidence and duration of ventricular tachycardias (VTs) and ventricular fibrillations (VFs). VF duration was recorded up until the time when BP <15 mmHg in rats that died with irreversible VF.

### Cardiac function evaluation

To assess cardiac function, invasive hemodynamic monitoring was performed. Heart rate, left ventricular systolic pressure (LVSP), maximal rates of LVSP (±dp/dt max) were continuously monitored using an electrophysiolograph (MH150; BioPAC Systems, Inc., Goleta, CA, USA).

### Moycardium injury assessment

Myocardium infarct size was measured as previously described^7^. 1% Evans Blue dye and 1% 2, 3, 5-triphenyltetrazolium chloride (pH 7.4) (both from Sigma-Aldrich; Merck KGaA) were used for myocardial staining. The stained myocardial slices were scanned (Epson v30, Seiko Epson Corporation, Nagano, Japan) and assessed using an image analysis system (Image-Pro Plus 3.0, Media Cybernetics). Creatine kinase-MB (CK-MB) and lactate dehydrogenase (LDH) were used as the specific indicator of acute myocardial injury. Blood samples were collected at the end of myocardium reperfusion and then centrifuged at 1,200 rpm for 20 min. The serum was collected to measure CK-MB (catalogue no. E006-1-1) and LDH (catalogue no. A020-2-2) using commercial kits (both from Jiancheng Bio, Nanjing, China), according to the manufacturer's instruction.

### Oxidative stress detection

Myocardial tissue and H9c2 cells were homogenized and centrifuged (2.400 rpm for 15 min at 4°C) to obtain the supernatants. The activity of superoxide dismutase (SOD) was detected using SOD assay kit (catalogue no. A001-1; Jiancheng Bio, Nanjing, China). Cardiac-free 15-F2t-isoprostane was used as a special indicator of oxidative stress-induced lipid peroxidation in myocardium injury^5^, which was detected using an ELISA kit (Cayman Chemical, Michigan, USA), according to the manufacturer's protocol.

### Transmission electron microscopy (TEM)

Observing autophagosomes under a TEM is a direct qualitative method of autophagy. At the end of reperfusion, 1 mm^3^ tissue samples from left ventricle were collected and fixed in a solution of 2.5% glutaraldehyde and 1% osmium tetroxide, dehydrated in an ascending series of alcohols and embedded in epoxy resin. Then, the stained slides were photographed under a TEM (HT7700, HITACHI, Tokyo, Japan).

### Study in H9c2 cell lines

H9c2 cells (American Type Culture Collection, Manassas, VA, USA) were cultured in Dulbecco's modified Eagle's medium (Gibco, MA, USA) containing 10% fetal bovine serum (Gibco, MA, USA) and 100 μg/ml penicillin/streptomycin in an atmosphere containing 5% CO_2_ at 37°C. When the cells' density reached 80%, they were trypsinized by 0.05% trypsin/1 mM EDTA (HyClone, USA) and plated onto 6-well culture plates (10^5^ cells /well) for experimental treatments. H9c2 cells were randomly divided into 7 groups: 1, low glucose (5.5 mM) medium (LG); 2, LG + hypoxia/reoxygenation (HR); 3, high glucose (30 mM) medium (HG); 4, HG + HR; 5, HG + HR + GDF11; 6, HG + HR + GDF11 + hydrogen peroxide(H_2_O_2_); 7, HG + HR + GDF11 + 3-Methyladenine(3-MA). GDF-11 (100mM) was given 1 h prior to hypoxia^13^, 3-MA (10 nM) (Selleck, chemicals, Houston, TX, USA) was treated at the beginning of hypoxia^6^ ^,^ ^7^. Cells in HR group went through 4 h of hypoxia followed by 2 h of reoxygenation. Hypoxic conditions were obtained using a gas incubator (5% CO2 and 95% N2). 3-MA (10 nM) or the vehicle DMSO at nontoxic concentrations had no effect on morphology or cell viability of H9c2 cells. Each experiment was performed more than 3 times independently in triplicate.

### Cell viability and oxidative stress assay

Cell viability and LDH were determined using Cell Counting Kit-8 (CCK-8) assay kit (catalogue no. 04-11; Dojindo, Kumamoto, Japan) and cytotoxicity assay kit (catalogue no. A020-2-2; Jiancheng, Nanjing, China), according to the manufacturer's protocols. Cells SOD and 15-F2t-isoprostane releases which reflect oxidative stress level were detected using assay kits according to the manufacturer's protocols.

### Western blot analysis

Western blotting was performed as described previously^7^. Antibodies against GAPDH (catalogue no. 2118), microtubule associated protein 1 light chain 3 β/α (LC3B/A, catalogue no. 12741), nuclear pore glycoprotein p62 (p62, catalogue no. 5114) (all from Cell Signaling Technology, Inc., Danvers, MA, USA) were used. Signals were detected using an Odyssey fluorescence imaging scanner and quantified using Odyssey software v3.0.29 (both from LI-COR, Lincoln, USA).

### Statistical analysis

Data are presented as the mean ± standard deviation. Differences among experimental groups were analyzed by one-way ANOVA or two-way ANOVA followed by a Bonferroni post-hoc test. *P*<0.05 was considered to be statistically significant. Analysis was performed by Prism software 7.0 (GraphPad Software, CA, USA).

## Results

### Pretreatment with GDF11 improved cardiac function and reduced myocadium injury in diabetic rats

As shown in Table 1, there was no significant difference in body weight and blood glucose among all the rats prior to diabetes induction. After STZ injection, diabetic rats presented notable diabetic symptoms. Compared with the age-matched non-diabetic subjects, rats with 8 weeks of diabetes exhibited decreased body weight and increased blood glucose.

**Table 1:** Body weights and blood glucose levels of non-diabetic and diabetic rats 8 weeks after STZ induction.

Basic parameters	Non-diabetic	Diabetic
Weight (g)	403.4 ± 18.62	*215.3 ± 9.08
Blood glucose (mmol/l)	5.27 ± 0.98	*22.36 ± 4.39

Data are presented as the means ± standard deviation (n=20 per group).

* P≤ 0.01 *vs.* non-diabetic rats.'

During MIR, the arrhythmias and mortality in diabetic were significantly higher than those in non-diabetic. GDF11 reduced IR-induced mortality, incidence of VT and VF in diabetic rats, but data did not reach significance; however, VT and VF durations were notably shorten, respectively, by pretreatment with GDF11 (Table 2).

**Table 2:** Effects of GDF11 on arrhythmias and mortality in diabetes induced by myocardial IR injury.

	Ventricular tachycardia		Ventricular fibrillation		Mortality (%)
	Incidence (%)	Duration (s)	Incidence (%)	Duration (s)	
N+IR	60	23.3 ± 6.0	40	56.9 ±16.0	11
D+IR	*86	*50.1 ± 15.0	*55	*91.1± 16.0	*31
GDF11+D+IR	79	#28.2 ± 8.0	51	#62.8 ± 17.1	28

Data are presented as the means ± standard deviation (n=12~14 per group).

* P≤ 0.05 *vs.* N+IR group,

# P≤ 0.05 *vs.* D+IR group. N, non-diabetes; D, diabetes; IR, ischemia reperfusion.'

Hemodynamic parameters were collected and analyzed to reflect left ventricular function. Diabetic rats showed a marked decrease in HR, LVSP, +dP/dt and -dP/dt compared with non-diabetic rats at baseline (Data not shown). As presented in Table 3, following 2h reperfusion, all of the hemodynamic parameters decreased in the diabetic and non-diabetic groups; the decrease in diabetic was more obvious. Treatment with GDF11 increased the levels of HR, LVSP, +dP/dt and -dP/dt in diabetic rats.

**Table 3:** Hemodynamic parameters of left ventricular function.

	HR (bpm)	LVSP (mmHg)	+dP/dt (mmHg/s)	-dP/dt (mmHg/s)
N+S	395 ± 29	124 ± 20	6402 ± 302	4307 ± 303
N+IR	*282 ± 30	*70 ± 8	*3331 ± 216	*2545 ± 407
D+S	*332 ± 31	*97 ± 15	*5338 ± 336	*3158 ± 217
D+IR	# &214 ± 40	# &45 ± 13	# &2389 ± 388	# *1667 ± 263
GDF11+D+IR	+306 ± 24	+64 ± 15	+3172± 307	+2239 ± 282

Data are presented as the means ± standard deviation (n=8~12 per group).

* P≤ 0.05 *vs.* N+S group,

# P≤ 0.05 *vs.* N+IR group,

& P≤ 0.05 *vs.* D+S group,

+ P≤ 0.05 *vs.* D+IR group. N, non-diabetes; D, diabetes; S, sham; IR, ischemia reperfusion; HR, heart rate; LVSP, left ventricular systolic pressure; dP/dt, change in LVSP.

**Figure 1:**
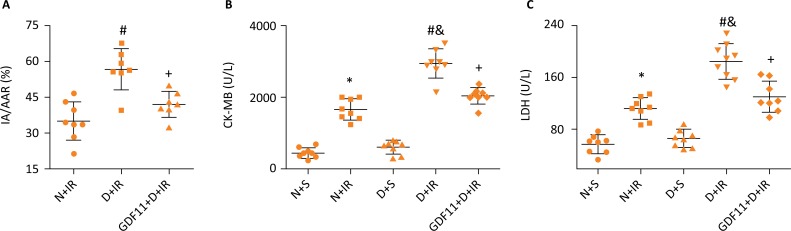
Effects of GDF11 on myocardial infarct size, CK-MB and LDH releases following 30 min ischemia followed by 2 h reperfusion, in non-diabetic and diabetic rats. **(A)** Percentage of area at risk vs. left ventricle. Biomarkers of the degree of injury **(B)** CK-MB and **(C)** LDH release. n=6-8/group. *P≤ 0.05 *vs.* N+S group, ^#^P≤ 0.05 *vs.* N+IR group, ^&^P≤ 0.05 *vs.* D+S group, ^+^P≤ 0.05 *vs.* D+IR group. N, non-diabetes; D, diabetes; S, sham; IR, ischemia reperfusion; IA/AAR, infarct area/area at risk; CK-MB, creatine kinase MB; LDH, lactate dehydrogenase.

Diabetic rats exhibited a significant increase in post-ischemia myocardial infarct size, CK-MB and LDH releases than non-diabetic rats. After treatment with GDF11, those 3 indicators were remarkably decreased, which reflected that GDF11 could reduce myocardial IR injury in STZ-induced type I diabetic rats (Fig. 1).

### GDF11 provided cardioprotection by antioxidant stress and up-regulation cardiac autophagy level in diabetic rats

**Figure 2:**
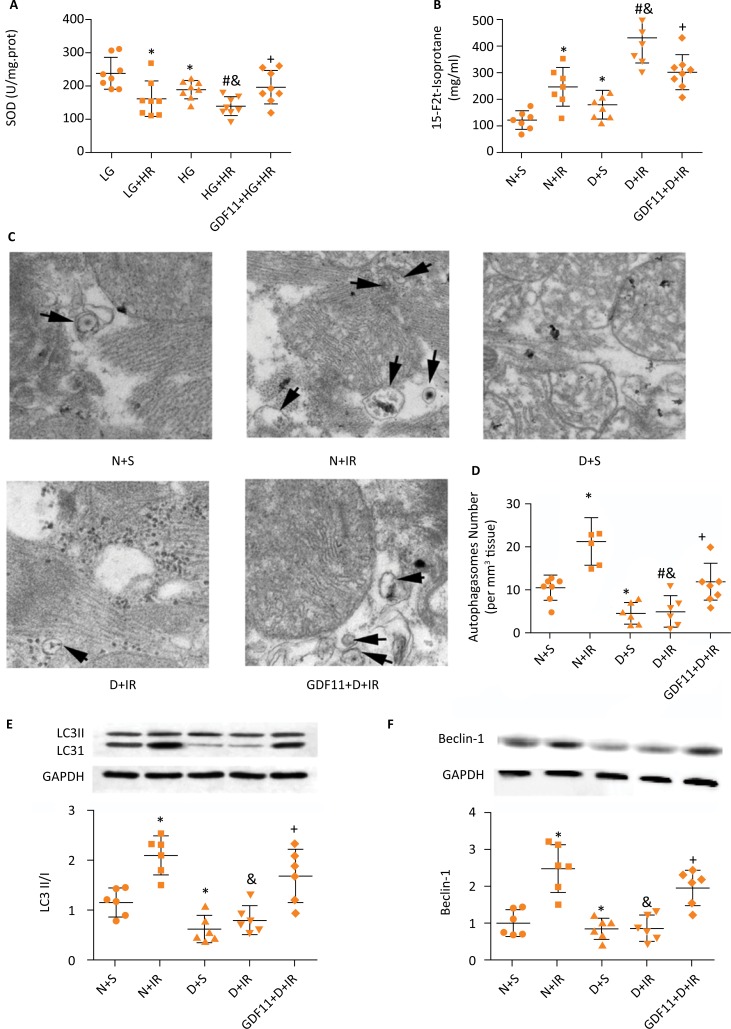
GDF11 provided cardioprotection by antioxidant stress and up-regulation cardiac autophagy level in diabetic rats. Biomarkers of the degree of oxidative stress **(A, B)** SOD activity and 15-F2t-isoprostane level were assayed, **(C, D)** autophagic vacuoles number,**(E)** LC3II/I ratio and **(F)** Beclin-1 level in diabetic myocardium was detected to reflect autophagy level. n=6-8/group. *P≤ 0.05 *vs.* N+S group, ^#^P≤ 0.05 *vs.* N+IR group, ^&^P≤ 0.05 *vs.* D+S group, ^+^P≤ 0.05 *vs.* D+IR group. N, non-diabetes; D, diabetes; S, sham; IR, ischemia reperfusion; SOD, superoxide dismutase.

After 2h reperfusion, diabetic myocardium exhibited lower SOD activity and higher 15-F2t-isoprostane level than non-diabetic myocardium. Pretreatment with exogenous GDF11 protein significantly elevated SOD activity and decreased 15-F2t-isoprostane level in diabetic IR myocadium, which reflected that the oxidative stress was effectively ameliorated (Fig. 2 A, B).

Then, we measured the myocardium autophagy level induced by the above processes. Observing autophagic vacuoles number via TEM and detecting autophagy-related protein are direct qualitative means for assessing autophagy. As shown in Figure 2 C-F, autophagic vacuoles number, LC3II/I ratio and Beclin-1 level in diabetic myocardium significantly decreased compared to non-diabetic myocardium at baseline. IR significantly increased autophagic vacuoles number, LC3II/I ratio and Beclin-1 level in non-diabetic rats, but not in diabetic rats. However, pretreatment with GDF11 significantly elevated autophagic vacuoles number, LC3II/I ratio and Beclin-1 level in diabetic myocardium following IR insult, indicating that GDF11 effectively enhanced autophagy level in diabetic hearts.

### GDF11 reduced HR injury in H9c2 cells exposed to HG condition

**Figure 3:**
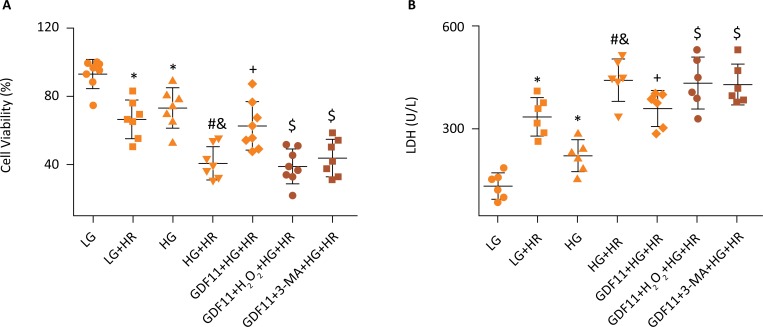
Effects of GAF11 on H9c2 cells that suffered HR injury in HG or LG conditions. **(A)** Results of CCK-8 assay and **(B)** LDH activity were analyzed. The results are representative of ≥3 independent experiments. *P≤ 0.05 *vs.* LG group, ^#^P≤ 0.05 *vs.* LG+HR group, ^&^P≤ 0.05 *vs.* HG group, ^+^P≤ 0.05 *vs.* HG+HR group, ^$^P≤ 0.05 *vs.* GDF11+HG+HR group. LG, low glucose medium; HG, high glucose medium; HR, hypoxia reoxygenation; 3-MA, 3-Methyladenine; CCK-8, cell counting kit-8; LDH, lactate dehydrogenase.

Additional investigations were implemented using embryonic rat cardiomyocyte derived H9c2 cells. H9c2 cells were exposed to HG conditions for 48h to simulate the diabetic myocardium. We observed that HG noticeably led to decreased cell viability and increased LDH release compared with LG group. These effects were further amplified by HR treatment. However, GDF11 administration significantly reduced HR injury in H9c2 cells exposed to HG conditions, as demonstrated by increased cell viability and decreased LDH release (Fig. 3). To further verify the function of GDF11 in the above process 3-MA, the autophagy inhibitor was administrated after GDF11 pretreatment, and our results showed that the protections provided by GDF11 were totally reversed by treatment with 3-MA. Notably, administration of the osmotic control mannitol did not affect the degree of cell injury in this experiment (data not shown).

### GDF11 provided protection against HR injury in H9c2 cells exposed to HG by antioxidant stress, elevating autophagy

**Figure 4:**
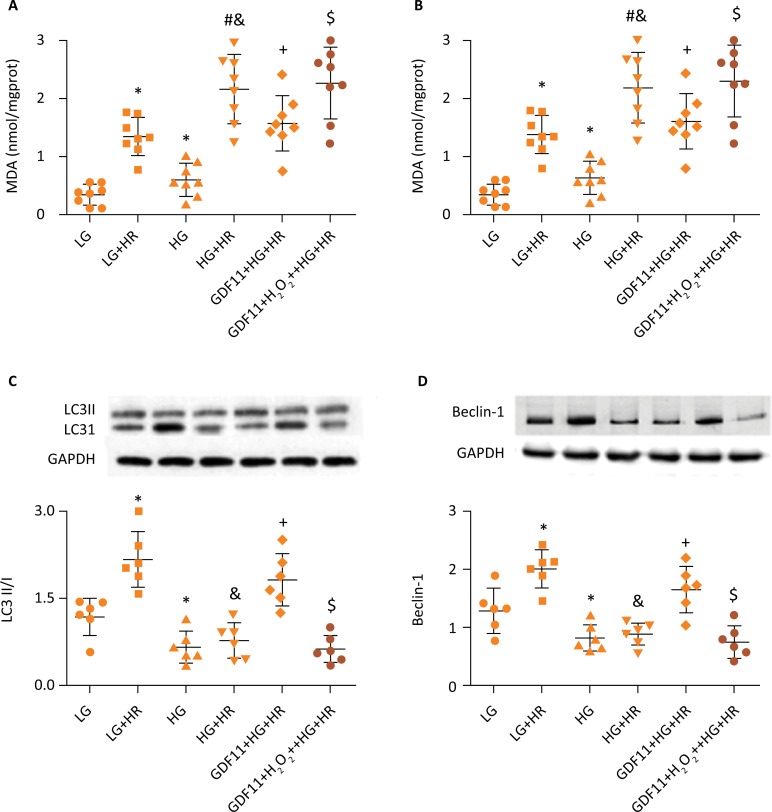
GDF11 provided protection against HR injury in H9c2 cells exposed to HG by antioxidant stress and elevating autophagy. **(A)** SOD activity, **(B)** LDH activity, **(C)** the ratio of LC3II/I and **(D)** Beclin-1 expression were analyzed. The results are representative of ≥3 independent experiments. *P≤ 0.05 *vs.* LG group, ^#^P≤ 0.05 *vs.* LG+HR group, ^&^P≤ 0.05 *vs.* HG group, ^+^P≤ 0.05 *vs.* HG+HR group, ^$^P≤ 0.05 *vs.* GDF11+HG+HR group. LG, low glucose medium; HG, high glucose medium; HR, hypoxia reoxygenation; 3-MA, 3-Methyladenine; SOD, superoxide dismutase; MDA, malondialdehyde.

As presented in Figure 4, pretreatment with GDF11 significantly decreased oxidative stress in H9c2 cells exposed to HG and HR injury, as evidenced by an increased SOD activity and decreased 15-F2t-isoprostane release. In addition, we observed that LC3II/I ratio and Beclin-1 level also increased following GDF11 pretreatment, reflecting that GDF11 can effectively restore autophagy activity simultaneously. In additional, induction of autophagic responses was significantly attenuated by 3-MA in this process.

## Discussion

The present study demonstrated that exogenous GDF11 provides cardioprotective effects against MIR in STZ-induced type 1 diabetic rats. Our results indicated that pretreatment with GDF11 remarkably improved cardiac function and reduced post-ischemia myocardial infarct size, which may be implicated in antioxidant stress and up-regulating autophagy level in diabetic myocardium. To the best of our knowledge, this is the first study to investigate the effects of GDF11 on IR injury in diabetic myocardium and hyperglycemic cardiomyocytes, and to explore the roles of oxidative stress and autophagy in this process.

Evidences from clinical and experimental studies have verified that diabetic hearts are more vulnerable to IR injury^15^ ^–^ ^17^. Aggravated oxidative stress induced by hyperglycemia, hyperlipidemia and insulin resistance in diabetic subjects was considered as the reason why diabetic myocardium cannot readily adapt to IR injury, thus, antioxidant stress is an effective method of defense against MIR injury in diabetes^15^ ^–^ ^17^. However, available methods are rare and cardioprotective effects are poor in clinical practice, presently. Therefore, it is quite necessary to explore the underlying mechanism of MIR in diabetes, and develop new therapeutic strategies for cardioprotection. In our present study, STZ-induced type 1 diabetic rats following 8 weeks of diabetes were employed, and a worse cardiac function accompanied by increased oxidative stress was observed in diabetic rats, compared with non-diabetic ones, which is in accordance with our previously studies^4^ ^–^ ^7^. After suffering MIR, diabetic rats showed more severe impairment of myocardial function and myocardium injury degree, as was evidenced from increased myocardial infarct size, higher levels of LDH and CK-MB and decreased levels in HR, MAP, ±dP/dt. Our results emphasized that diabetes remarkably aggravates myocardium IR injury, which is consistent with the results of previous studies^5^ ^–^ ^6^.

GDF11, also named bone morphogenetic protein (BMP11), is a member of the activin transforming growth factor β(TGF-β) superfamily. GDF11 is widely expressed in tissues and organs^8^, and it exerts its biological effects mainly by binding to the TGFβ type I and II receptors, then motivating the smad2/3 signaling^8^. GDF11 plays key roles in embryonic development, bone and muscle formation^20^ ^,^ ^21^, and it was also reported as an anti-aging factor in previously studies. Old mice that received GDF11 treatment achieved the same benefits as those that received heterochronic parabiosis, specifically in the reversal of age-related cardiac hypertrophy^8^. Thus, it appears to preserve the regenerative ability of stem cells and rejuvenate the function of multiple organs in old mice^8^. In 2017, Du *et al*.^23^ reported that ultrasound targeted microbubble destruction (UTMD) mediated delivery of GDF11, rejuvenating the aged mouse heart and enhancing chronic cardiac function and cellular regeneration after acute IR injury. Then, it was demonstrated that GDF11 provides cardioprotective effects by enhancing cellular self-protection or self-repair. A newly study focused on myocardial IR injury found that exogenous GDF11 significantly reduced post-ischemia myocardium infarct size and may have morphologic and functional roles in the recovery in the early stage of IR, which implicated in reduction of oxidation and inflammation via attenuating non-canonical TGF-β signaling^13^. However, whether GDF11 could confer protective effects to MIR in diabetes, and its underling mechanisms are still unclear.

To verify the scientific problem above, diabetic rats were daily treated with exogenous GDF11 for 4w, as previously described^23^. In our study, pretreatment with GDF11 significantly improved cardiac morphologic and function in diabetic rats; it also decreased arrhythmia severity, the post-ischemia myocardial infarct size, CK-MB and LDH release, which strongly indicated that GDF11 proved cardiac function during IR and conferred protective effects against MIR injury. Further studies were performed to explored the underlying mechanisms. Data from our *in vivo* and *in vitro* studies showed that GDF11 ameliorated MIR injury, accompanied by the increase of cardiac SOD activity and the decrease of 15-F2t-isoprostane release. They also showed that oxidative stress was notably suppressed in this process, and that GDF11 provides cardioprotective effects by antioxidant stress, and perhaps, this is consistent with a recent study performed by Su *et al*. ^13^and Anqi *et al*.^24^.

Interestingly, the results in our further study showed that autophagy activation was implicated in the decrease of MIR injury by pretreatment with GDF11 in diabetic rats, simultaneously. Autophagy is a dynamic and evolutionarily conserved catabolic process which is comprised of autophagosome formation and autolysosomal clearance that targets damaged or dysfunctional organelles to the lysosome for degradation and recycling; it is an important and indispensable mechanism in cellular metabolism and survival^25^. Autophagy is essential for cardiac homeostasis, and it may be activated in response to a stressful condition; excessive autophagy leads to programmed cell death^26^. Observing autophagosome under TME is recognized as the golden standard for autophagy level assessment; the conversion of LC3A to LC3B is a symbol of autophagosome formation. p62/sequestome 1 protein is able to bind ubiquitinated cargo designated for autophagic breakdown, and was observed to reflect myocardial autophagy status; thus, it is an improved marker of autophagic flux for measuring the number and morphology of autophagosomes, ratio of LC3B/LC3A and levels of p62. Autophagy participated in the pathological process of IR injury. Organ ischemia forcefully activates the autophagic response as a protective strategy, and autophagy is sharply up-regulated during reperfusion^26^. Autophagy excessiveness or insufficiency is detrimental to organs subjected to IR injury; therefore, maintaining autophagy at a proper level is necessary. In our previous studies, we observed that autophagy was totally suppressed in myocardium from STZ-induced type 1 diabetic rats; it may be responsible for the increased venerability to IR injury and the loss of ischemia post conditioning-induced cardioprotection. Pretreatment with autophagy inducer rapamycine significantly reduced the degree of myocardial IR injury in diabetic hearts^6^ ^,^ ^7^. In our present study, autophagy inhibition was also observed in diabetic myocardium and H9c2 cells with HG exposure, a finding consistent with those of previous studies^6^. The autophagic response was not activated by IR or HR injury in *in vivo* and *in vitro* studies, respectively. After treatment with exogenous GDF11 protein, we found that the autophagy level in myocardium and H9c2 cells was significantly increased by IR or HR insults, as evidenced by the increased level of autophagosomes number, high ratio of LC3B/LC3A and increased levels of Beclin1 expression. The data strongly indicated that the protective effects provided by GDF11 may be involved in autophagy up-regulation.

To further verify the underlying mechanism of the cardioprotection provided by GDF11, an additional study was performed in H2c2 cells. H_2_O_2_, a strong oxidant, and 3-MA, the autophagy inhibitor, were administrated after GDF11 pretreatment, respectively. Our data demonstrated that the protective effects provided by GDF11 in H9c2 cells with HG exposure were completely abolished by H_2_O_2_ and 3-MA, strongly suggesting that the cardioprotecive effects by GDF11 was implicated in antioxidant stress and autophagy up-regulation.

Taken together, the findings of our present study provide compelling evidence that exogenous recombinant GDF11 contributes to morphologic and functional recovery after myocardial IR injury in STZ-induced type 1 diabetic rats. The cardioprotective effects provided by GDF11 are implicated in antioxidant stress and up-regulation autophagy during IR injury. Therefore, according to our evidence, GDF11 shows potential as a novel therapy for reducing the vulnerability of MIR in diabetes, while, further studies are needed to explore the underlying molecular mechanisms.

## Conclusion

GDF11 contributes to morphologic and functional recovery after MIR injury in diabetic rats, and may be implicated in antioxidant stress and up-regulation autophagy during IR injury.
